# Effects of stem cells from inducible brown adipose tissue on diet-induced obesity in mice

**DOI:** 10.1038/s41598-021-93224-6

**Published:** 2021-07-06

**Authors:** Enrique Calvo, Noelia Keiran, Catalina Núñez-Roa, Elsa Maymó-Masip, Miriam Ejarque, Joan Sabadell-Basallote, María del Mar Rodríguez-Peña, Victòria Ceperuelo-Mallafré, Jesús Seco, Ester Benaiges, Theodora Michalopoulou, Rosa Jorba, Joan Vendrell, Sonia Fernández-Veledo

**Affiliations:** 1 Servei D’Endocrinologia I Nutrició I Unitat de Recerca, Hospital Universitari de Tarragona Joan XXIII, Institut D’Investigació Sanitària Pere Virgili (IISPV), c/ Dr. Mallafré Guasch 4, 43007 Tarragona, Spain; 2 CIBER de Diabetes Y Enfermedades Metabólicas Asociadas (CIBERDEM) - Instituto de Salud Carlos III, Madrid, Spain; 3 Universitat Rovira I Virgili, Tarragona, Spain; 4 Servei de Cirurgia General I de L’Aparell Digestiu, Hospital Universitari Joan XXIII, Institut D’Investigació Sanitària Pere Virgili (IISPV), Tarragona, Spain

**Keywords:** Physiology, Stem cells, Diseases, Medical research

## Abstract

Adipose-derived mesenchymal stem cells (ASCs) are a promising option for the treatment of obesity and its metabolic co-morbidities. Despite the recent identification of brown adipose tissue (BAT) as a potential target in the management of obesity, the use of ASCs isolated from BAT as a therapy for patients with obesity has not yet been explored. Metabolic activation of BAT has been shown to have not only thermogenic effects, but it also triggers the secretion of factors that confer protection against obesity. Herein, we isolated and characterized ASCs from the visceral adipose tissue surrounding a pheochromocytoma (IB-hASCs), a model of inducible BAT in humans. We then compared the anti-obesity properties of IB-hASCs and human ASCs isolated from visceral white adipose tissue (W-hASCs) in a murine model of diet-induced obesity. We found that both ASC therapies mitigated the metabolic abnormalities of obesity to a similar extent, including reducing weight gain and improving glucose tolerance. However, infusion of IB-hASCs was superior to W-hASCs in suppressing lipogenic and inflammatory markers, as well as preserving insulin secretion. Our findings provide evidence for the metabolic benefits of visceral ASC infusion and support further studies on IB-hASCs as a therapeutic option for obesity-related comorbidities.

## Introduction

The continuing epidemic of obesity points to an urgent need for the development of new therapeutic approaches focused on prevention and treatment of this pathological disorder and its related co-morbidities. Regenerative cell therapies based on mesenchymal stem cells (MSCs) are an alternative to traditional clinical strategies^1^, and are being widely explored for the treatment of many disorders, including obesity, by virtue of their high self-renewal capacity and ability to differentiate into multiple stromal cell lineages. MSC-based therapies might also be a promising tool in the management of systemic metabolic syndromes, as they have demonstrated abilities to mobilize and regulate inflammation and immunity in a paracrine manner, in large part through the release of chemokines, cytokines and extracellular vesicles at sites of injury^2^.

MSCs were initially isolated from the bone marrow^3^, but are now known to reside in a broad variety of tissues^4^. Adipose tissue is a major source of MSCs, as adipose-derived stem cells (ASCs) can be easily isolated from the stromal-vascular fraction of adipose tissue, which is abundantly available and can be harvested using minimally invasive techniques (e.g., liposuction)^5–7^. The potential of human ASCs in the treatment of type 2 diabetes mellitus and its related metabolic disorders has been recently explored in pre-clinical models, with encouraging results. While the mechanism of action is not completely understood, infusion of autologous or heterologous ASCs (from human subcutaneous white adipose tissue [scWAT]) in rodent models of obesity has been shown to significantly suppress body-weight gain and to improve dyslipidemia and glucose tolerance^8–12^.

Considering this recent evidence, and based on the well-known involvement of brown adipose tissue (BAT) in systemic energy metabolism and its protective actions against obesity and diabetes^13^, we wondered whether infusion of human ASCs isolated from BAT could also mitigate the metabolic abnormalities associated with diet-induced obesity (DIO) in mice. A caveat to the isolation and study of ASCs from classical human BAT is its scarcity in adults. Indeed, remnants are only found in the neck, mediastinum, axilla, retroperitoneum and abdominal wall^14^, and tend to be small and surgically challenging to obtain through biopsy. To circumvent these experimental limitations, here we isolated ASCs from the ectopic brown periadrenal adipose tissue surrounding a pheochromocytoma, which is considered as a model of inducible BAT in humans^15^. Pheochromocytoma is an uncommon neuroendocrine tumor arising from the adrenal medulla and is characterized by elevated secretion of catecholamines, which are key drivers of WAT browning^16,17^.

In the present study, we explored and compared the potential anti-obesity and anti-diabetic effects of ASCs isolated from human inducible BAT (IB-hASCs) with those of ASCs isolated from visceral WAT (W-hASCs). We found that infusion of IB-hASCs in a DIO rodent model reduced body weight and improved glucose tolerance similarly to W-hASCs, but had superior anti-lipogenic and anti-inflammatory effects in adipose tissue. These beneficial effects were concomitant with a greater capacity of IB-hASCs to preserve glucose-stimulated insulin secretion in an obese setting. An exploratory proteomics analysis revealed that the ASCs displayed differences in their secretory profile. Overall, our results suggest that visceral and IB-hASCs might be a promising research avenue to explore for future obesity therapies.

## Results

### IB-hASCs express stem cell and browning markers

**Figure 1:**
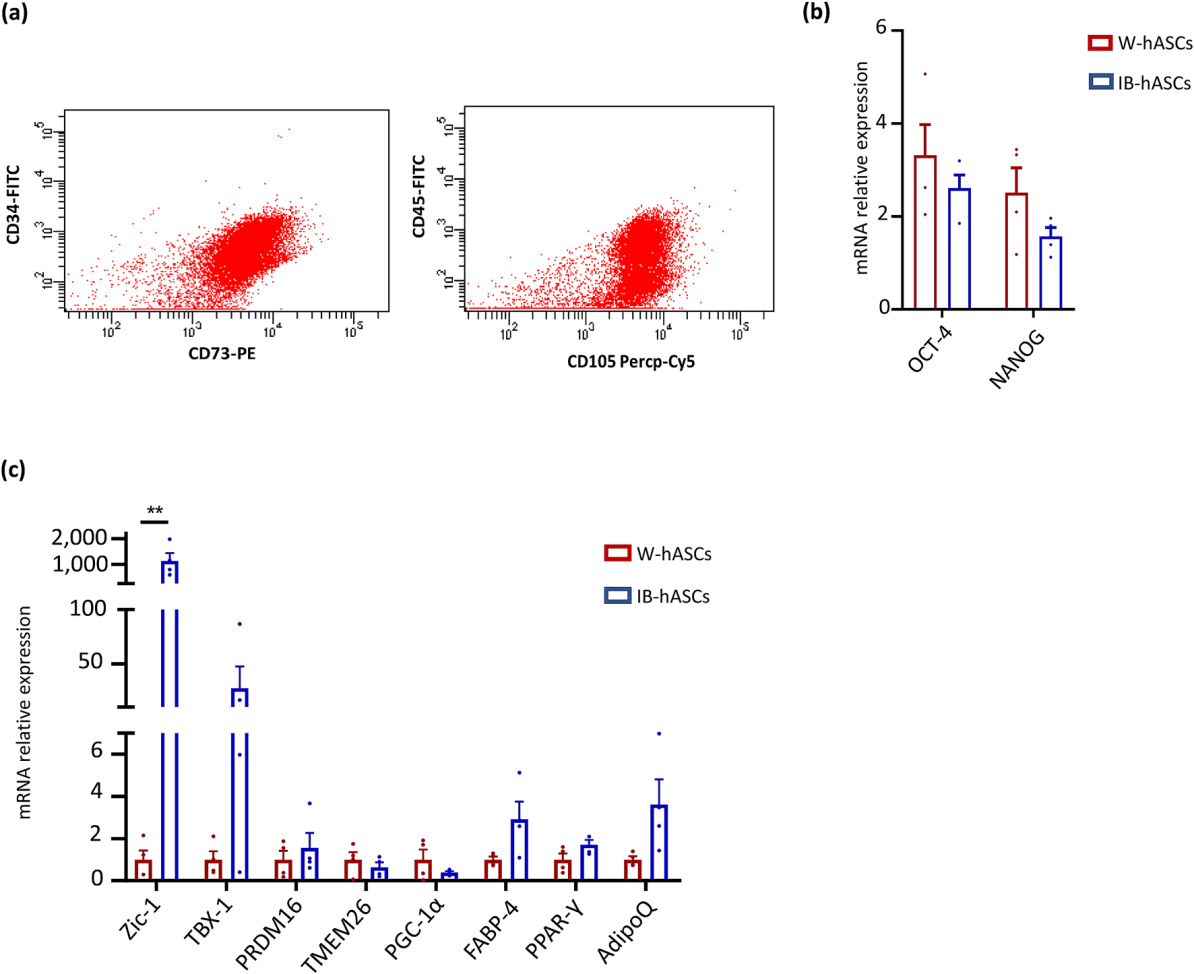
Characterization of stem cells isolated from adipose tissue surrounding a pheochromocytoma. (**a**) Representative flow cytometry analysis confirming that induced brown (IB)-hASCs are positive for the mesenchymal stem cell markers CD73 and CD105. (**b**) Expression of stem cell markers OCT-4 and NANOG in IB-hASCs and in W-hASCs isolated from vWAT. (**c**) Expression of brown-brite and adipocyte markers in IB-hASCs and in W-hASCs from vWAT. n = 4 per group (independent donors). Data are shown as mean ± s.e.m. **P < 0.01 (unpaired t-test).

According to the International Society for Cellular Therapy (ISCT), human MSCs are defined as plastic-adherent, positive for CD90, CD105, CD73 markers, negative for CD45, CD34, CD14, CD19 and MHC II, and with the ability to differentiate into cells of mesodermal origin such as osteoblasts, chondroblasts and adipocytes^18^. Flow cytometry analysis confirmed the mesenchymal phenotype of isolated IB-hASCs (Fig. 1a). Additionally, we surveyed the expression of the stem cell markers *OCT4* and *NANOG* in IB-hASCs, finding that the expression was similar to that observed for the well-characterized W-hASCs from visceral WAT (Fig. 1b).

We also quantified the expression of browning (*ZIC1, TBX1, PRDM16, TMEM26* and *PGC1α*) and adipocyte (*PPARγ, FABP4 and ADIPOQ*) markers in W-hASCs and IB-hASCs isolated from 8 different patients (4 per group). As shown in Fig. 1c, the expression of *ZIC1* was significantly higher in IB-hASCs than in W-hASCs and there was a trend for higher expression of *TBX1*. No significant differences in adipocyte markers were observed between the two cell types.

### Infusion of IB-hASCs or W-hASCs reduces weight gain and improves glucose tolerance in mice with diet-induced obesity

**Figure 2:**
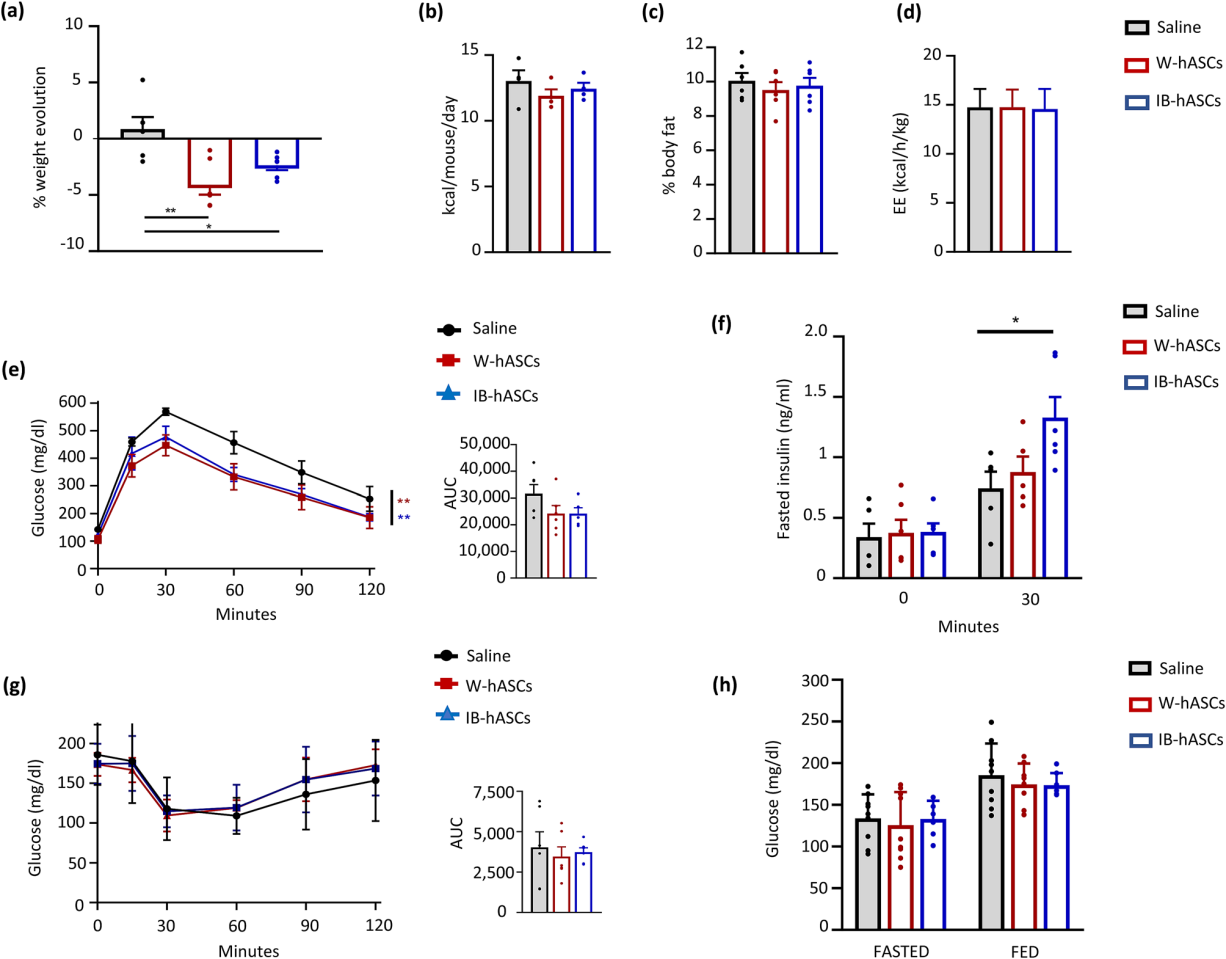
Infusion of IB-hASCs suppresses body-weight gain and improves glucose tolerance in DIO mice. (**a**) Body-weight progression and (**b**) food intake of high-fat diet-treated mice infused with W-hASCs, IB-hASCs or saline (n = 6). (**c**) Percentage of body fat content. (**d**) Energy expenditure. (**e**) Glucose tolerance test (n = 6) and area under the curve (AUC) analysis. (**f**) Insulin tolerance test (n = 5–6) and AUC analysis. (**h**) Fed and fasted plasma glucose levels. Results are expressed as mean ± s.e.m. *P < 0.05, **P < 0.01 (two-way ANOVA; one-way ANOVA).

The anti-obesity effects of human ASCs from scWAT administered in DIO rodents is well recognized^8,9,11^. We found that several of the metabolically beneficial effects reported for the aforementioned cells were also observed with hASCs isolated from vWAT. For instance, compared with the saline control group, DIO mice infused with W-hASCs or IB-hASCs showed significant weight loss over the three weeks of high-fat diet (HFD) feeding (Fig. 2a). No significant differences were observed in food intake (Fig. 2b), body fat content (Fig. 2c) or energy expenditure (Fig. 2d) between the three groups. The weight reduction in ASC-treated DIO mice was accompanied by a significant improvement in glucose tolerance measured by a glucose tolerance test (Fig. 2e). The improved glucose tolerance in DIO mice infused with IB-hASCs was likely related to the significant increase in insulin secretion measured 30 min after glucose infusion (0.74 ± 0.137 ng/ml saline vs. 1.32 ± 0.171 ng/ml IB-hASCs, *P* = 0.014) (Fig. 2f), an effect not observed in mice treated with W-hASCs. However, no differences were observed in insulin sensitivity between the three groups of DIO mice, as measured by an insulin tolerance test (Fig. 2g). Likewise, no between-group differences were found for fed or fasted plasma glucose levels (Fig. 2h). Evaluation of the homing capacity of the ASCs by flow cytometry detection of CD90 revealed that intravenously-injected cells accumulated mainly in the lung 14 h after injection, but also migrated to other organs, especially vWAT and also spleen and liver (Supplementary Fig. S1 online).

### IB-hASC and W-hASC therapy modulates lipid metabolism and induce adipose tissue browning in mice with diet-induced obesity

**Figure 3:**
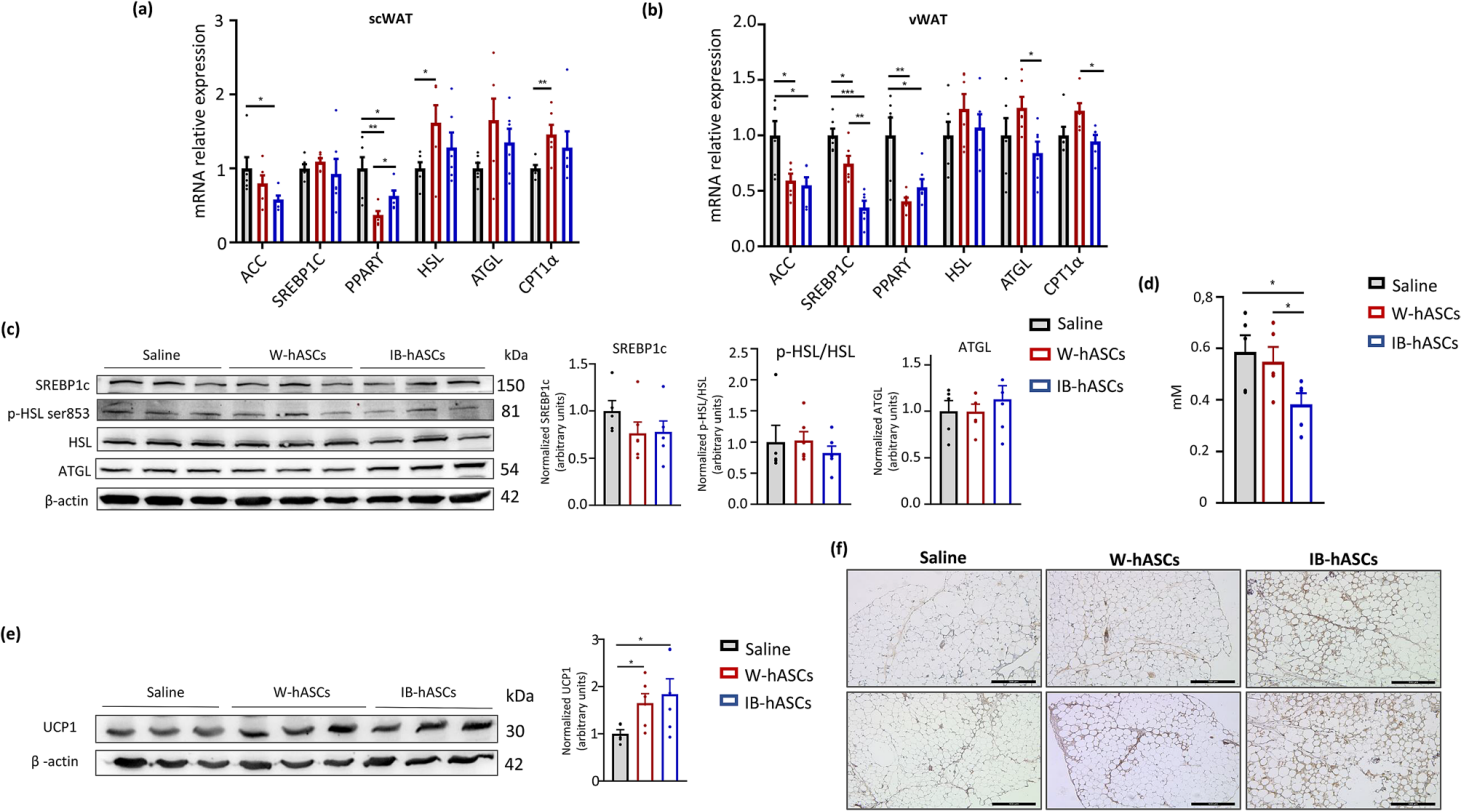
Infusion of IB-hASCs decrease the expression of lipogenic genes and the levels of circulating free fatty acids, and triggers Ucp1 expression in adipose tissue of DIO mice. Mean mRNA expression levels of adipogenic/lipolytic genes in (**a**) scWAT and (**b**) vWAT. (**c**) Representative western blot analysis for phospho-Hsl (ser853), Hsl, Atgl and β-actin in vWAT. (**d**) Quantification of fasting serum free fatty acids. (**e**) Representative western blot analysis for Ucp1 in vWAT. (**f**) Immunohistochemistry for Ucp1 in representative sections of vWAT. Scale bar = 500 µm. (n = 5–6 for saline and n = 6 for W-hASCs and IB-hASCs). Data are shown as mean ± s.e.m. *P < 0.05, **P < 0.01, ***P < 0.001 (one-way ANOVA).

The coupling of lipogenesis, lipolysis and browning in adipose tissue plays a critical role in regulating energy balance and homeostasis. Accordingly, we next surveyed the expression of genes involved in lipid metabolism in the adipose tissue of DIO mice treated with saline, W-hASCs or IB-hASCs. Analysis of the scWAT and vWAT compartments revealed that the expression of the lipogenic genes *Acc*, *Pparγ* and *Srebp-1c* in vWAT was lower in the two groups of ASC-treated mice than in the control (saline) group, and the decrease was greater for *Acc* and *Srebp-1c* in scWAT and vWAT, respectively, in mice infused with IB-hASCs (Fig. 3a,b). By contrast, the expression of *Ppar*γ in scWAT and *Cpt1α* (lipolysis-related gene) in vWAT was consistently higher in W-hASC-infused mice than in IB-hASC- or saline-infused mice (Fig. 3a,b). Western blot analysis showed a tendency for a decrease in the levels of Srebp-1c in ASC-treated mice, whereas no differences were observed for phosphorylated-Hsl or Atgl protein expression between groups (Fig. 3c). Of note, only the infusion of IB-hASCs resulted in a significant decrease in the levels of circulating free fatty acids (FFAs) in DIO mice (Fig. 3d).

We also analyzed the protein expression of Ucp1 in vWAT as a direct marker of browning. Results showed that vWAT Ucp1 levels analyzed both by western blot (Fig. 3e) and immunohistochemistry (Fig. 3f) were greater in both W-hASC- and IB-hASC-treated DIO mice than in equivalent saline-treated mice (Fig. 3e), demonstrating that infusion of ASCs of visceral origin promotes browning of adipose tissue.

### Infusion of IB-hASC or W-hASCs reduces inflammatory gene expression in adipose tissue of mice with diet-induced obesity

**Figure 4:**
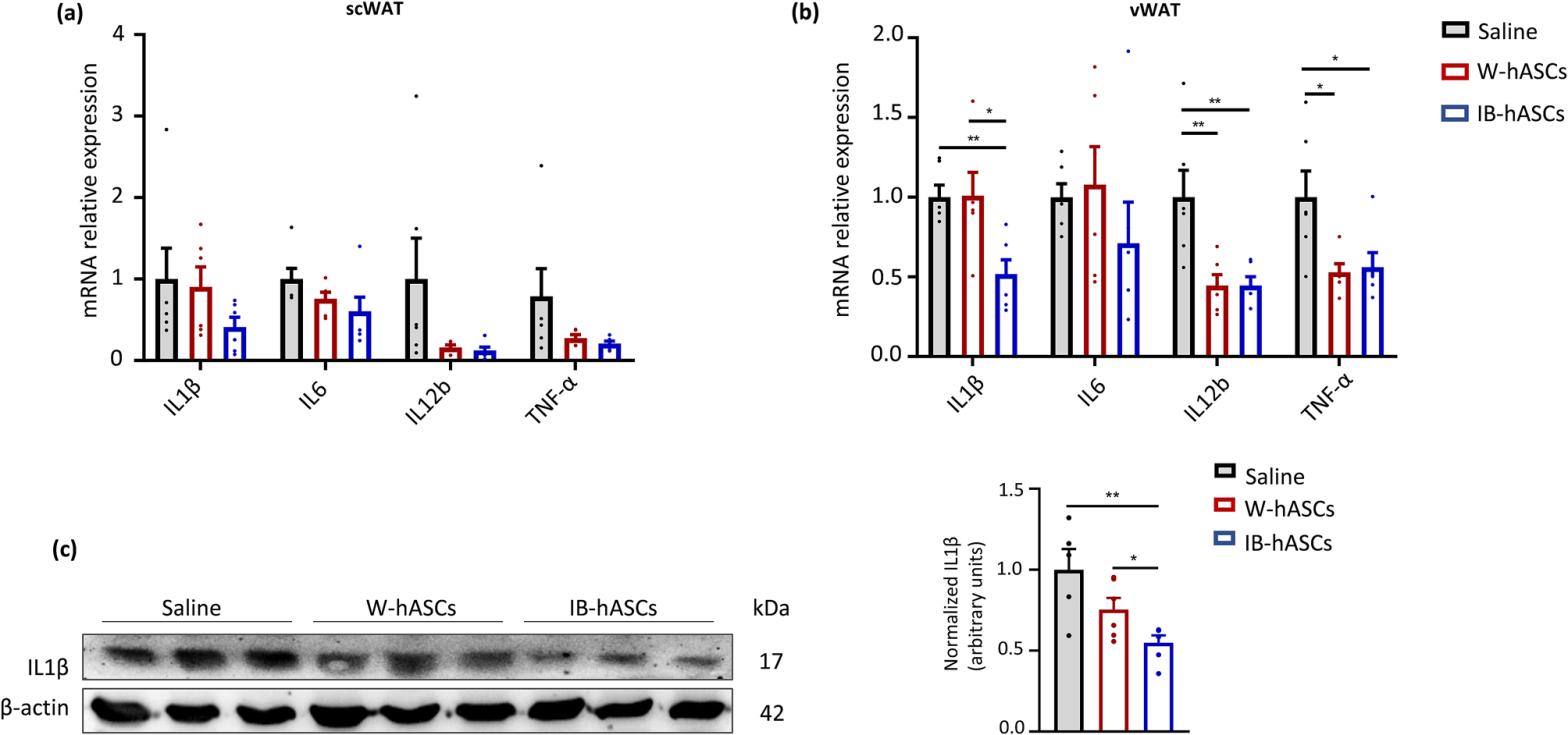
Infusion of IB-hASCs ameliorates adipose tissue inflammation. (**a**) Mean mRNA expression levels of pro-inflammatory cytokines in scWAT and (**b**) vWAT (n = 6). (**c**) Representative western blot analysis for IL1ß and β-actin in vWAT (n = 5 for saline; n = 6 for W-hASCs and IB-hASCs). Data are shown as mean ± s.e.m. *P < 0.05, **P < 0.01 (one-way ANOVA).

Obesity is known to be associated with systemic low-grade chronic inflammation, which likely plays a causal role in obesity-related metabolic complications. One of the main sites of inflammation is adipose tissue, which acts as an endocrine organ by secreting cytokines that affect energy metabolism and the inflammatory response. To further confirm the beneficial effects of IB-hASC therapy on obesity, we examined the gene expression profile of several pro-inflammatory cytokines (*Il1ß*, *Il6*, *I12* and *Tnfα*) in both scWAT and vWAT depots of DIO mice treated with saline, W-hASCs or IB-hASCs. While no significant changes in the expression of these pro-inflammatory markers were found in the scWAT compartment (Fig. 4a), treatment with W-hASCs and IB-hASCs significantly reduced *Il12* and *Tnfα* expression in vWAT (Fig. 4b). Moreover, mice treated with IB-hASCs showed a reduction in *Il1β* expression with respect to the other groups (Fig. 4b), and this was confirmed by western blotting as compared with the untreated saline group (Fig. 4c). Overall, our data uncover the protective anti-obesity and anti-inflammatory effects of both vWAT-derived hASCs and BAT-derived hASCs in a murine model of DIO, with the latter seeming to have superior effects.

## Discussion

Herein, we provide the first demonstration of the benefits of hASCs of visceral origin, including ASCs isolated from inducible BAT, to treat metabolic disorders linked to obesity in DIO mice, which provides support for their potential as a therapeutic option to target human obesity and associated disorders.

Pre-clinical studies testing subcutaneous-derived ASCs for obesity and related co-morbidities have demonstrated some beneficial effects in different rodent models of obesity and diabetes^8,9,11,12^. In this setting, adipose tissue appears to be a key responsive organ for ASC-related anti-obesity therapies through enhanced lipolysis and browning, and the inhibition of lipogenesis and stimulation of lipolysis have been linked to a reduction in adiposity^8^. Our data are partly in accord with this view as we found a trend for a decrease in adiposity in ASC-treated mice. Also, mRNA analysis of genes related to lipolysis, lipogenesis and β-oxidation in adipose tissue correlated with tendential albeit non-significant changes in SREBP, p-HSL and ATGL protein expression, and changes in circulating FFAs, particularly in the case of IB-hASC therapy.

We show for the first time to our knowledge that the administration of ASCs derived from both visceral WAT and inducible BAT attenuates weight gain and improves glucose tolerance even in the setting of HFD feeding. We found that infusion of IB-hASCs, but not W-hASCs, also diminished the level of circulating FFAs, which are associated with the changes observed in visceral and subcutaneous lipid metabolism in the case of IB-hASC therapy. It is well known that chronic exposure to FFAs causes peripheral (skeletal muscle) insulin resistance^19^, and also impairs pancreatic β-cell insulin secretion^20^. While we failed to find differences in insulin sensitivity between the three groups of mice, we observed that the IB-hASC-related improvement in glucose tolerance was concomitant with higher glucose-stimulated insulin secretion, supporting the notion that adipose tissue metabolism and pancreatic function are linked^21,22^.

We observed the induction of adipose tissue browning after visceral ASC therapy, in agreement with a previous report using scWAT-derived ASCs^8^. It is known that “browning” leads to heat production and increases energy expenditure, which might contribute to counteract obesity and to improve glucose and adipose tissue homeostasis^8,23,24^. We show that glucose tolerance is improved in ASC-treated mice, which correlated with increased adipose tissue Ucp1 protein expression. UCP1 is proposed to have functions beyond thermogenesis, as mice lacking this uncoupling protein do not develop an obese phenotype^25,26^. We found, however, that the induction of Ucp1 expression by IB-hASCs did not correlate with greater energy expenditure. Indeed, a recent human study revealed that energy expenditure is not associated with the expression of browning markers in adipose tissue^27^. Consequently, UCP1-independent mechanisms might be crucial in the regulation of energy balance.

Another interesting observation from our study was the change in the expression of anti-inflammatory genes associated with ASC infusion, particularly with IB-hASC treatment. An increase in adiposity due to overnutrition is known to provoke an initial inflammatory response to facilitate adipose tissue expansion, which in turn promotes angiogenesis to prevent hypoxia and induces insulin resistance to reduce the energy accumulation in cells^28,29^. The low state of inflammation in adipose tissue can trigger the release of pro-inflammatory adipokines which, via an intricate cascade, co-ordinately interact with immune cells to control the hormonal sensitivity of adipocytes^30,31^. The complexity of inflammation in obesity limits its management focused on the blockade of specific inflammatory targets^28^. Chronic inflammation modulates a plethora of immunologic and inflammatory mediators that impact insulin-targeting organs, leading ultimately to various metabolic disorders including insulin resistance^32–34^. Although the trigger for this inflammation remains unclear, there is an evident relationship between the degree of inflammation and the severity of obesity comorbidities^35^. The lipolytic actions of ASC-based therapies result in a breakdown of adipose lipid depots and a reduction in the secretion of adipocyte pro-inflammatory cytokines, which contributes to suppress the low-grade inflammation associated with obesity. Additionally, the anti-inflammatory effects of MSCs have been related to their interaction with macrophages, as MSCs secrete factors that attenuate the activation of pro-inflammatory (M1) macrophages and promote the switching of resident M1 cells to anti-inflammatory (M2) types^36^. Consequently, there is a systemic metabolic effect which, for example in pancreas, ameliorates the impairment in insulin secretion by blocking the islet cell apoptosis provoked by overexposure to pro-inflammatory cytokines^37,38^. In accord with these findings, we observed a reduction in the expression of inflammatory markers in scWAT and vWAT after IB-hASC infusion, and the conservation of insulin secretion.

The mechanisms underlying the therapeutic benefits of MSC therapy remain unclear. It has been suggested that the homing of these multipotent cells to damaged tissues stimulates their differentiation into specific functioning cells^9,11,39^. It is broadly accepted, however, that the beneficial effects of MSCs are mainly related to the release of endocrine and paracrine factors including chemokines, cytokines and microRNAs, collectively termed the secretome, which exerts immunomodulatory functions and drives the regulation of various physiological processes^40,41^. Our results revealed a tissue-specific homing when ASCs are injected, mainly to lungs, but also to vWAT and liver to a lesser extent (Supplementary Fig. S1 online). By contrast, scWAT did not show any presence of human ASCs after injection. Based on our results, it is plausible that the metabolic effects observed after ASC therapy may be attributed to paracrine mechanisms associated with their secretome.

The comparative analysis of the ASC secretome might provide new insights into candidate molecules participating in homeostatic functions^42–44^, and also might help to elucidate potential differential mechanisms of action between W-hASCs and IB-hASCs therapy. In this sense, it is known that ASCs from human BAT and WAT exhibit different metabolic activities^45^ and, at the tissue level, the secretory profile of WAT differs from that of BAT^46^. The concept of BAT as a secretory organ has recently emerged based on the abundant regulatory molecules it releases, the so-called brown adipokines, which target different peripheral tissues and the central nervous system. These adipokines not only trigger the activation of the thermogenic function of BAT, but can also be considered as modulators of systemic metabolism^47,48^. Specifically, factors derived from activated BAT such as fibroblast growth factor 21 and interleukin-6 (IL-6), among others, have been found to target adipose tissue and pancreas, where they stimulate white-fat browning and likewise contribute to better glucose homeostasis by improving β-cell function^47,48^.

Despite the differences between the secretory characteristics of both types of adipose tissue, and considering that the molecular profiles of MSC secretomes are tissue-specific^49^, as far as we know no studies have specifically analyzed the secretome of ASCs from BAT. Our preliminary analysis of the hASC protein secretome using an untargeted approach with liquid chromatography coupled to tandem mass spectrometry revealed significant differences in several factors secreted by hASCs isolated from the adipose tissue surrounding a pheochromocytoma compared with those isolated from visceral adipose tissue from lean and healthy subjects (Supplementary Fig. S2 online). Interestingly, among the highly secreted proteins differentially expressed by IB-hASCs, we identified titin, a protein traditionally linked to muscular contraction and to shivering thermogenesis, which have also been implicated in non-shivering thermogenesis by impacting on BAT activity^50^. Members of the Serpin family (Serpine2), which are associated with adipose tissue insulin sensitization^51^, and several extracellular matrix (ECM)-related factors such as aggrecan, TIAM-2 and collagen alpha-1 (VIII) were also significantly more secreted by IB-hASCs. Our results are in agreement with previous findings describing ECM-related proteins as specific brown adipokines potentially involved in the adaptation of BAT ECM to thermogenic functions^52,53^.

By contrast, the secreted proteins found to be up-regulated in W-hASCs were mainly related to Golgi structural maintenance (i.e., golgin subfamily A members 3 and putative golgin subfamily A member 8I). Unexpectedly, the secretion of IL-6, a cytokine released by activated BAT important for thermogenesis, was greater in W-hASC cultures than in IB-hASC cultures, which suggests that differences in tissue secretomes might differ according to cell type and context. Further research will be needed to decipher the function and impact of these secretory factors.

Overall, our present findings, supported by previous studies, suggest that ASCs of visceral origin, including IB-hASCs, should be further explored to clarify their specific mechanisms and their potential as therapeutic agents in the context of metabolic disease.

## Material and methods

### Adipose tissue stem cell isolation and culture

ASCs were isolated from peri-tumoral adipose tissue (named IB-hASCs) obtained during surgical procedures of patients diagnosed with a pheochromocytoma (2 males, 2 females; mean age 58.8 years); and from visceral adipose tissue (named W-hASCs) from healthy donors (1 male, 3 females; mean age 50.2 years) obtained during scheduled non-acute minor surgical interventions. Patients were recruited at the University Hospital Joan XXIII (Tarragona, Spain) in accordance with the tenets of the Declaration of Helsinki. The study was approved by the Ethics and Research Committee of the Institut d’Investigació Sanitària Pere Virgili (IISPV CEIm, Tarragona, Spain), and all participants signed a written informed consent before the study.

hASCs were isolated as described^54,55^. Briefly, adipose tissue was washed with phosphate buffered saline (PBS) and then treated with 0.1% collagenase in PBS + 1% bovine serum albumin (BSA) for 1 h at 37 °C with gentle agitation. Digested samples were centrifuged at 300 × *g* at 4 °C for 5 min to separate stromal cells. The stromal pellet fraction was resuspended in DMEM/F12, 10% fetal bovine serum (FBS) and 1% antibiotic/antimycotic solution. To prevent spontaneous differentiation, primary cultures of ASCs at passage 0 (P0) were grown to 90% confluence and then harvested with trypsin–EDTA.

### Flow cytometry

Collected stromal vascular cells were cultured in stromal culture medium, consisting of DMEM/F12, 10% FBS, and 1% penicillin/streptomycin, at 37 °C overnight. P0 cultures were grown to 90% confluence, washed in PBS and then detached with trypsin for immunophenotypic analysis^56^. Cells were incubated with a combination of the following fluorochrome-conjugated monoclonal antibodies: FITC anti-CD34 (clone 581), PerCP-Cy5.5 anti-105 (clone 266), FITC anti-CD45 (clone HI30) and PE anti-CD73 (clone AD2) (all from BD Pharmingen, San José, CA) for 20 min at room temperature (protected from light). Data were acquired on a FACS Aria III flow cytometer and were analyzed using FACSDiva software (both from BD Biosciences, San José, CA).

### Animals

C57BL/6J male mice (7-weeks-old) were purchased from Charles River Laboratories (Barcelona, Spain) and were maintained in a controlled environment under a 12-h light–dark cycle with ad libitum access to food and water. After a 1-week acclimatization period, the diet was switched from standard chow (8.4% kcal from fat [A04]; SAFE diets, Augy, France) to HFD (45% kcal from fat [D12451]; Research Diets Inc., New Brunswick, NJ) for 16 weeks to provoke DIO. At the age of 24 weeks, DIO mice were randomly assigned to three groups: (a) IB-hASC infusion, (b) W-hASC (from vWAT) infusion and (c) control, saline infusion. Mice were infused once weekly for three weeks with 2 × 10^5^ cells (from pools of 4 different donors) or with saline, through the tail vein. MSCs lack major histocompatibility complex (MHC) II, leading to the inactivation of T-cells, and possess immunosuppressive properties that confer a characteristic non-immunogenic profile, allowing safe allogeneic cell transplantation without immunosuppression^57^.

Mice continued on the HFD regimen until sacrifice. Body weight and food intake were measured weekly. After the three weeks of therapy, glucose tolerance and insulin tolerance tests were performed, after which the mice were sacrificed for adipose tissue and blood analysis. Animal studies were carried out in accordance with ARRIVE guidelines. All animal care procedures were supervised and approved by the Universitat Rovira i Virgili Animal Welfare and Ethics Committee, and conformed to European Union Directive 86/609/EEC and recommendation 2007/526/EC regarding the protection of animals used for scientific purposes, enacted under Spanish law 1201/2005.

### ASC homing

Additional groups of mice (three for each condition) were used to assess the homing of injected ASCs. To do this, mice were infused with a single tail vein injection of 2 × 10^5^ cells or saline and were sacrificed 14 h later. Organs were then extracted and homing was examined in the peripheral blood, spleen, liver, lungs, scWAT and vWAT. Individual cells from the different organs were obtained after digestion in 0.1% collagenase (as described above) for 1 h and 30 min at 37 °C with gentle agitation. Digested tissues were passed through a 70-μm strainer (Fisher Scientific, Waltham, MA). In the case of peripheral blood, we used the 1 × Lyse lysing solution (BD Pharmingen, Oxford, UK) to lyse red blood cells. In total, 1 × 10^7^ cells were used for staining with a phycoerythrin-conjugated CD90 monoclonal antibody (Clone 5E10, BD Pharmingen) after preincubation with FcR blocking reagent (Miltenyi Biotec, Bergisch Gladbach, Germany). Cells obtained from non-transplanted mice stained with the same antibodies were used to exclude false positive cells and to establish the negative control. Data were acquired on FACSAria III flow cytometer (BD Biosciences) and were analyzed using FACSDiva v8 (BD Biosciences) and FlowJo v10 (Ashland, OR) software.

### Glucose and insulin tolerance tests

Glucose and insulin tolerance tests were performed as reported previously^32^. For the glucose tolerance test, an intraperitoneal injection of glucose solution (1.5 g glucose/kg body weight) was administered after 16 h of fasting. Glucose was measured from tail vein punctures at 0, 15, 30, 60, 90 and 120 min using a handheld glucometer (Accu-Chek glucose reader; Roche, Mannheim, Germany). To measure insulin levels in response to glucose, blood was collected from tail punctures at 0 and 30 min post-injection. Insulin was measured using a commercial mouse insulin ultra-sensitive enzyme-linked immunosorbent assay (BioVendor Research and Design products, Brno, Czech Republic). For intraperitoneal insulin tolerance analysis, mice were fasted for 3 h and were then injected with insulin (0.75 U/kg), and glucose was measured as described above.

### Free fatty acid determination

Serum FFAs were determined using an FFA Kit (MAK044, Sigma-Aldrich, St. Louis, MO).

### Indirect calorimetry

Energy expenditure (kcal h^−1^ kg^−1^) was calculated using a MM-100 Metabolic Monitor (CWE Incorporated, Ardmore, PA) from the amount of O_2_ consumed and the amount of CO_2_ produced (ml h^−1^ kg^−1^) using the following equation: energy expenditure (J) = 15.818*V*O_2_ + 5.176*V*CO_2_.

### Ucp1 immunohistochemistry

Immunohistochemistry was done as described by Escalona-Garrido and colleagues^58^. Paraffin sections were heated for 20 min in 100 mM sodium citric buffer (pH 6.0) and 0.05% Tween-20 and then blocked for 10 min with a solution of 9% H_2_O_2_ in 10% methanol. To block non-specific binding, sections were incubated with 6% BSA and 2% normal horse serum in PBS-0.1% Triton X-100 for 1 h at room temperature. Incubation with the primary antibody (1:500 UCP1, ab10983; Abcam Cambridge, UK) was performed overnight at 4 °C. Tissue sections were then washed in PBS followed by incubation with a biotinylated secondary anti-rabbit antibody (1:250, BA-1100; Vector Laboratories Inc., Burlingame, CA) for 1 h at room temperature. The slides were washed with PBS and stained with DAB-immunoperoxidase (SK-4100; Vector Laboratories). After a final wash with water, samples were counterstained with hematoxylin (H3136, Sigma-Aldrich) solution for 10 s and dehydrated through xylene incubation. Finally, the sections were mounted with Depex (18,243.02, SERVA Electrophoresis GmbH, Heidelberg, Germany) and dried overnight. Images were viewed using a Leica DM4 B microscope.

### Gene expression analysis

As previously described^32^, total RNA from tissues was isolated using the RNeasy Lipid Tissue Mini Kit (Qiagen Science, Hilden, Germany) and converted to cDNA using the Reverse Transcription System (Applied Biosystems, Carlsbad, CA). Quantitative gene expression was analyzed by real-time PCR (RT-PCR) on a 7900HT Fast Real-Time PCR System using the TaqMan Gene Expression Assay (Applied Biosystems) (Supplementary Table S1 online). Fold differences were calculated using the comparative Ct method (2^−ΔΔCt^), and were expressed relative to the expression of the housekeeping gene 18S.

### Immunoblot analysis

Thirty micrograms of scWAT and vWAT protein were resolved by SDS-PAGE electrophoresis, transferred to PVDF membranes and blocked in 5% non-fat milk in T-TBS. Samples were subjected to immunoblot analysis with polyclonal antibodies against SREBP-1c, ATGL (Cell Signaling Technologies, Danvers, MA), UCP1, phospho-HSL ser853 (equivalent to ser 563) (Abcam) and β-actin (Sigma-Aldrich), which was used as a loading control. Immunoreactive bands were detected using the SuperSignal West Femto chemiluminescent substrate (Pierce Biotechnology, Rockford, IL). Western blot signals were quantified using Quantity One software (Bio-Rad, Hercules, CA).

### Statistical analysis

In vitro data are expressed as mean ± s.e.m. Differences between two groups were determined using an unpaired t-test (two-tailed, 95% confidence interval). One-way analysis of variance (ANOVA) was conducted to examine differences between three groups. Statistical significance of glucose and insulin tolerance tests were tested using two-way ANOVA. A P-value < 0.05 was considered statistically significant. All the statistical analyses were performed using GraphPad Prism 6.0 software (GraphPad Inc., San Diego, CA).

## Supplementary Information

Supplementary Information.

## Data Availability

The datasets generated during and/or analyzed during the current study are available from the corresponding author on reasonable request.
