# Supplementary material for: Effects of stem cells from inducible brown adipose tissue on diet-induced obesity in mice
Source: Sci Rep. 2021 Jul 6;11:13923. doi: 10.1038/s41598-021-93224-6 (PMC8260805; doi:10.1038/s41598-021-93224-6)
Supplement: Supplementary file 1 — Supplementary Information. [file 41598_2021_93224_MOESM1_ESM.pdf]

Effects of stem cells from inducible brown adipose tissue on diet-induced obesity in mice

Enrique Calvo[1,2], Noelia Keiran[1,2,3], Catalina Núñez-Roa[1,2], Elsa Maymó-Masip[1,2], Miriam Ejarque[1,2], Joan Sabadell-Basallote[1,2,3], María del Mar Rodríguez[1,2], Victòria Ceperuelo-Mallafré[1,2], Jesús Seco, [1,2], Ester Benaiges[1,2,3], Theodora Michalopoulou[1], Rosa Jorba[4], Joan Vendrell[1,2,3]*, Sonia Fernández-Veledo[1,2]*

Suppl. Table S1. Mice gene expression Taqman probes. Results were calculated using the comparative Ct method and expressed relative to the expression of the housekeeping gene 18S.

Detector	Taqman probes	Gene probes
**Stem cell markers**		
NANOG	Hs02387400_m1	NANOG homeobox
OCT-4	Hs01570480_m1	Octamer-binding transcription factor
**Inflammatory markers**		
IL1b	Mm00434228_m1	Interleukin 1 Beta
IL12b	Mm22818759_m1	Interleukin 12 Beta
IL6	Mm00446190_m1	Interleukin 6
TNF-α	Mm00443258_m1	Tumor necrosis factor alpha
**Lipid metabolism genes**		
PPARγ	Hs01115513_m1<br>Mm00440940_m1	Peroxixome proliferator-activated receptor gamma
FABP4	Hs01086177_m1	Fatty acid-binding protein 4
ADIPOQ	Hs00605917_m1	Adiponectin
HSL	Mm00495359_m1	Hormone-sensitive lipase
SREBP-1c	Mm00550338_m1	Sterol regulatory element-binding transcription factor 1
ACC	Mm01304257_m1	Acetyl-CoA carboxylase
ATGL	Mm00503040_m1	Adipose Triglyceride Lipase
CPT1α	Mm1843037_m1	Carnitine palmitoyltransferase 1
**Browning genes**		
TMEM26	Hs00415619_m1	Transmembrane Protein 26
TBX1	Hs00962558_g1	T-box 1
PRDM16	Hs00223161_m1	PR-domain containing 16
ZIC1	Hs00602749_m1	Zinc finger transcription factor 1
PGC1α	Hs00173304_m1	Peroxisome proliferator-activated receptor-gamma coactivator 1- alpha
**Housekeeping**		
18S rRNA	Mm03928990_g1	18S ribosomal RNA

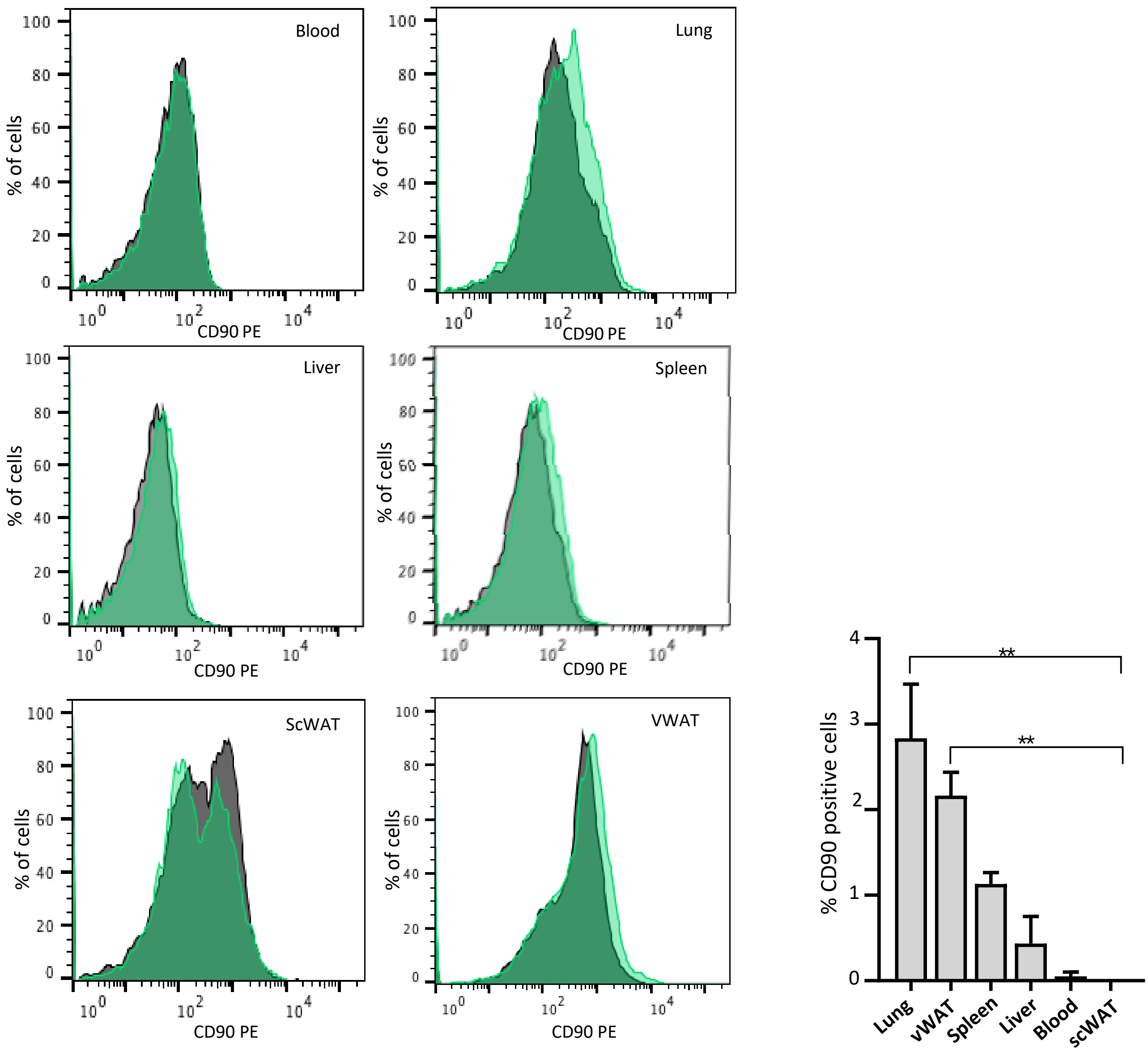

Suppl. Fig. S1. ASCs injection mainly accumulate in lung and vWAT. Percentage of CD90 positive cells in lung, vWAT, spleen, liver, blood and scWAT. Results are expressed as mean ± s.e.m.; ***P*<0.01, (one-way ANOVA).

Regarding the “chopping” of some immunoblots, we would like to clarify that we design each experiment so that we can perform the analysis of as many parameters as possible on precisely the very same membrane in order to avoid repeated stripping (thus minimizing stripping-derived interferences in Western Blot signals), and also to diminish loading-derived differences since we test multiple proteins in exactly the same lane. This implies that sometimes each membrane is blotted with several antibodies at the same time when this is allowed by the electrophoretical migration of the bands that are to be studied. The subsequent digital acquisition of the image is sometimes performed on the whole membrane but, for the sake of clarity, the image is then subdivided in as many sections as required for the number of proteins analyzed (thereby the “chopping” of the final image shown in the figure). Almost every membrane is treated in this way for the sake of reliability and reproducibility. In the case of p-HSL/HSL, the membrane was stripped and reproved. Also note that different membranes are labeled as Mb1, Mb2, Mb3, Mb4 and Mb5.

Full unedited gels for Figure 3C

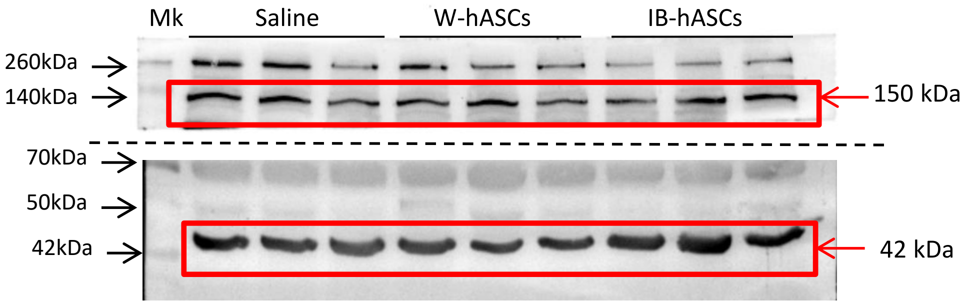

Membrane 1 → vWAT
Upper membrane: anti-SREBP-1c
Lower membrane: anti-β-actin

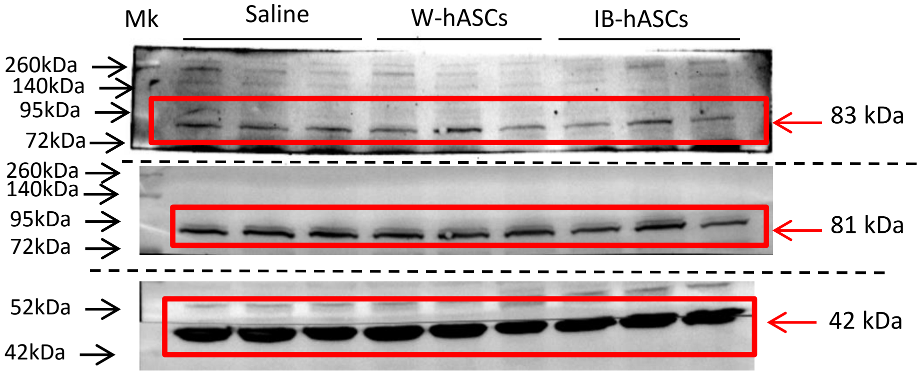

Membrane 2 → vWAT
Upper membrane: anti-pHSL
Median membrane: anti-HSL
Lower membrane: anti-β-actin

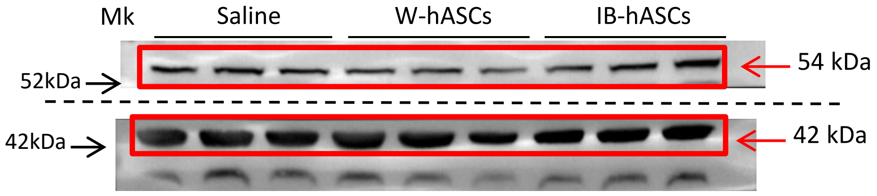

Membrane 3 → vWAT
Upper membrane: anti-ATGL
Lower membrane: anti-β-actin

Full unedited gels for Figure 3E

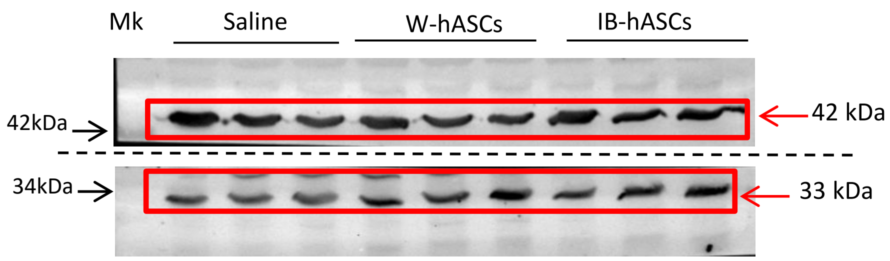

Membrane 4 → vWAT
Upper membrane: anti-β-actin
Lower membrane: anti-UCP1

Full unedited gels for Figure 4C

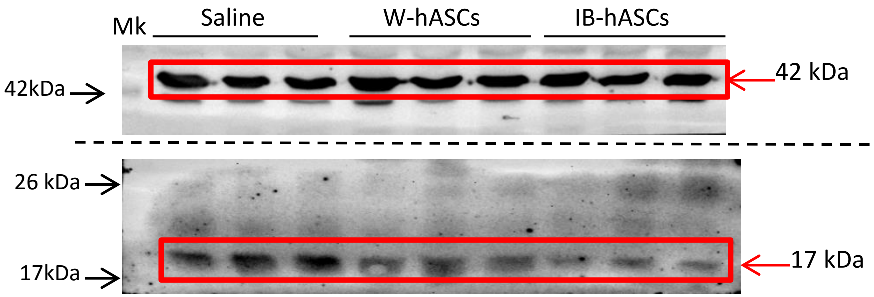

Membrane 5 → vWAT
Upper membrane: anti-β-actin
Lower membrane: anti-IL1β

(a)

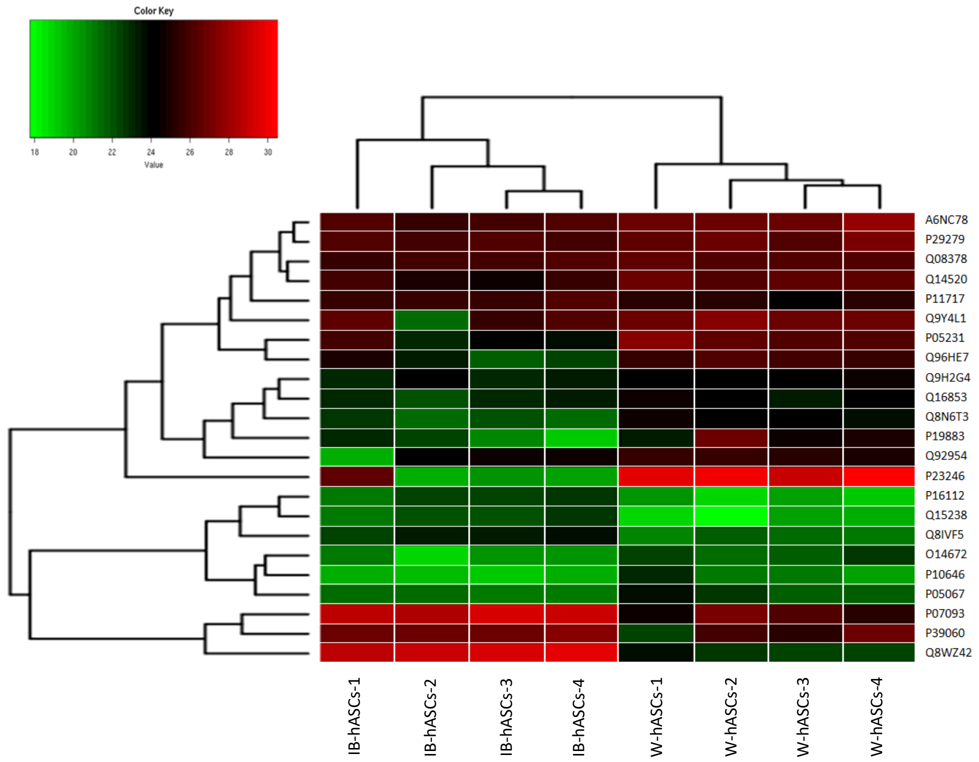

(b)

Protein ID	Description	GO biological process
A6NC78	**Putative golgin subfamily A member 8I [GOLGA8IP]**	Golgi organization
P29279	**CCN family member 2 [CNN2]**	Cell adhesion, extracellular matrix constituent secretion, cell migration
Q08378	**Golgin subfamily A member 3 [GOLGA3]**	Spermatogenesis
Q14520	**Hyaluronan-binding protein 2 [HABP2]**	Cell adhesion, proteolysis
P11717	**Cation-independent mannose-6-phosphate receptor [IGF2R]**	Lysosomal transport, animal organ regeneration
Q9Y4L1	**Hypoxia up-regulated protein 1 [HYOU1]**	Endoplasmic reticulum to Golgi vesicle-mediated transport
P05231	**Interleukin-6 [IL6]**	Inflammatory response, glucose homeostasis,
Q96HE7	**ERO1-like protein alpha [ERO1A]**	Extracellular matrix organization
Q9H2G4	**Testis-specific Y-encoded-like protein 2 [TSPYL2]**	Negative regulation of cell cycle and cell growth
Q16853	**Membrane primary amine oxidase [AOC3]**	Cell adhesion, inflammatory response
Q8N6T3	**ADP-ribosylation factor GTPase-activating protein 1 [ARFGAP1]**	Endoplasmic reticulum to Golgi vesicle-mediated transport
P19883	**Follistatin [FST]**	Cell differentiation, multicellular organism development
Q92954	**Proteoglycan 4 [PRG4]**	Immune response
P23246	**Splicing factor, proline- and glutamine-rich [SFPQ]**	mRNA processing, innate immune response
P16112	**Aggrecan core protein [ACAN]**	Extracellular matrix organization, cell adhesion
Q15238	**Pregnancy-specific beta-1-glycoprotein 5 [PSG5]**	Female pregnancy
Q8IVF5	**T-lymphoma invasion and metastasis-inducing protein 2 [TIAM2]**	Activation of GTPase activity, positive regulation of apoptotic process
O14672	**Disintegrin and metalloproteinase domain-containing protein 10 [ADAM10]**	Extracellular matrix disassembly, positive regulation of cell growth and migration
P10646	**Tissue factor pathway inhibitor [TFP1]**	Blood coagulation
P05067	**Amyloid-beta precursor protein [APP]**	Extracellular matrix organization, positive regulation of mitotic cell cycle
P07093	**Glia-derived nexin [SERPINE2]**	Regulation of cell migration, secretion by cell
P39060	**Collagen alpha-1(XVIII) chain [COL18A1]**	Cell adhesion, extracellular matrix organization
Q8WZ42	**Titin [TTN]**	Muscle contraction, shivering and non-shivering thermogenesis

Suppl. Fig. S2. Analysis of hASC protein secretomes. Untargeted proteomics approach by liquid chromatography coupled to tandem mass spectrometry was used to identify and quantify the secreted factors from hASCs isolated from adipose tissue surrounding the pheochromocytoma (IB-hASCs 1–4) and from visceral adipose tissue from lean and healthy subjects (W-hASCs 1–4). A total of 3555 proteins were identified by proteomic analysis in 6-hour conditioned media of hASCs. After removal of conflictive protein entries, related to low quality or noisy mass spectrometry intensity peaks, and retaining only those proteins found in at least 4 patients, a total of 2927 proteins were filtered for statistical analysis. Protein expression data was log2-transformed to reduce skewness distributions. An unpaired Mann-Whitney test was used to identify a total of 23 high-to-medium quality protein entries with an adjusted p-value < 0.05 after Bonferroni correction (a). Table describing the secreted factors and their more relevant biological functions (b).

LIQUID CHROMATOGRAPHY-MASS SPECTROMETRY METHODS

Sample preparation

ASCs cultures in passage 6 were plated at a density of 12,000 cells/cm2 in 35 mm culture plates (Nunc, Denmark) and allowed to grow for 24 hours. The plates were firstly washed with PBS without Ca2+/Mg2+ (Invitrogen, USA), and next, stromal medium without FBS was added. After 9h, the conditioned media (CM) was collected and frozen at −80 °C until it was required.

400uL of CM samples were concentrated to 50uL in Amicon Ultra 3K columns (Milipore). Samples in 6M urea were then reduced with dithiothreitol (30 nmol, 37 ºC, 60 min) and alkylated in the dark with iodoacetamide (60 nmol, 25 ºC, 30 min). The resulting protein extract was first diluted to 2M urea with 200 mM ammonium bicarbonate for digestion with endoproteinase LysC (1:10 w:w, 37ºC, o/n, Wako, cat # 129-02541), and then diluted 2-fold with 200 mM ammonium bicarbonate for trypsin digestion (1:10 w:w, 37ºC, 8h, Promega cat # V5113). After digestion, peptide mix was acidified with formic acid and desalted with a MicroSpin C18 column (The Nest Group, Inc) prior to LC-MS/MS analysis.

LCMS analysis

Samples were analyzed using a LTQ-Orbitrap Fusion Lumos mass spectrometer (Thermo Fisher Scientific, San Jose, CA, USA) coupled to an EASY-nLC 1200 (Thermo Fisher Scientific (Proxeon), Odense, Denmark). Peptides were loaded directly onto the analytical column and were separated by reversed-phase chromatography using a 50-cm column with an inner diameter of 75 µm, packed with 2 µm C18 particles spectrometer (Thermo Scientific, San Jose, CA, USA).

Chromatographic gradients started at 95% buffer A and 5% buffer B with a flow rate of 300 nl/min for 5 minutes and gradually increased to 25% buffer B and 75% A in 79 min and then to 40% buffer B and 60% A in 11 min. After each analysis, the column was washed for 10 min with 10% buffer A and 90% buffer B. Buffer A: 0.1% formic acid in water. Buffer B: 0.1% formic acid in 80% acetonitrile.

The mass spectrometer was operated in positive ionization mode with nanospray voltage set at 2.4 kV and source temperature at 305°C. The acquisition was performed in data-dependent acquisition (DDA) mode and full MS scans with 1 micro scans at resolution of 120,000 were used over a mass range of m/z 350-1400 with detection in the Orbitrap mass analyzer. Auto gain control (AGC) was set to 4E5 and charge state filtering disqualifying singly charged peptides was activated. In each cycle of data-dependent acquisition analysis, following each survey scan, the most intense ions above a threshold ion count of 10000 were selected for fragmentation. The number of selected precursor ions for fragmentation was determined by the "Top Speed" acquisition algorithm and a dynamic exclusion of 60 seconds. Fragment ion spectra were produced via high-energy collision dissociation (HCD) at normalized collision energy of 28% and they were acquired in the ion trap mass analyzer. AGC was set to 2E4 and an isolation window of 0.7 m/z and a maximum injection time of 12 ms were used. All data were acquired with Xcalibur software.

Digested bovine serum albumin (New England biolabs cat # P8108S) was analyzed between each sample to avoid sample carryover and to assure stability of the instrument and QCloud[59] has been used to control instrument longitudinal performance during the project.

Data Analysis

Acquired spectra were analyzed using the Proteome Discoverer software suite (v2.3, Thermo Fisher Scientific) and the Mascot search engine (v2.6, Matrix Science) [60]. The data were searched against a Swiss-Prot human database plus a list of common contaminants and all the corresponding decoy entries [61]. For peptide identification a precursor ion mass tolerance of 7 ppm was used for MS1 level, trypsin was chosen as enzyme, and up to three missed cleavages were allowed. The fragment ion mass tolerance was set to 0.5 Da for MS2 spectra. Oxidation of methionine and N-terminal protein acetylation were used as variable modifications whereas carbamidomethylation on cysteines was set as a fixed modification. False discovery rate (FDR) in peptide identification was set to a maximum of 1%. Protein abundance was estimated as the average peak area of the 3 most intense peptides for a given protein.

Differential enrichment was done using DEP package that provides an integrated analysis workflow for robust and reproducible analysis of mass spectrometry proteomics data [62]. Briefly, identified proteins were filtered to keep those detected in all, or all but one, replicates of at least one condition, resulting in 23 proteins retained. The filtered data was then background corrected and normalized by variance stabilizing transformation [63]. Protein-wise linear models combined with empirical Bayes statistics were used for the differential enrichment analysis using limma package [64].

REFERENCES

59. Chiva, C. *et al*. QCloud: A cloud-based quality control system for mass spectrometry-based proteomics laboratories. *PLoS One* **11,** 13 (2018).

60. Perkins, D. N., Pappin, D. J., Creasy, D. M. & Cottrell, J. S. Probability-based protein

identification by searching sequence databases using mass spectrometry data. *Electrophoresis* **20,** 3551-3567 (1999).

61. Beer, L. A., *et al*. Efficient quantitative comparisons of plasma proteomes using label-free analysis with MaxQuant. *Methods Mol Biol*. **1619,** 339-352 (2017).

62. Zhang X., *et al*. Proteome-wide identification of ubiquitin interactions using UbIA-MS. *Nature Protocols* **13,** 530–550 (2018).

63. Välikangas et al. *Briefings in Bioinformatics*. https://academic.oup.com/bib/article/19/1/1/2562889 (2016).

64. Ritchie M.E., *et al*. "limma powers differential expression analyses for RNA-sequencing and microarray studies." *Nucleic Acids Research*, doi: 10.1093/nar/gkv007 (2015).
